# Assessment of the Mandibular Incisive Canal by Panoramic Radiograph and Cone-Beam Computed Tomography

**DOI:** 10.1155/2014/187085

**Published:** 2014-09-23

**Authors:** Ricardo Raitz, Elisabeth Shimura, Israel Chilvarquer, Marlene Fenyo-Pereira

**Affiliations:** ^1^Discipline of General Pathology, University of São Caetano do Sul (USCS), São Caetano do Sul, SP, Brazil; ^2^São Leopoldo Mandic Dental Research Center, Campinas, SP, Brazil; ^3^Instituto de Documentação Ortodôntica e Radiodiagnóstico (INDOR), São Paulo, SP, Brazil; ^4^Discipline of Radiology, Department of Stomatology, School of Dentistry, University of São Paulo (FOUSP), São Paulo, SP, Brazil

## Abstract

*Objectives.* The region between mental foramens is considered as a zone of choice for implants. However, complications may arise due to an extension anterior to the mental foramen that forms the mandible incisive canal [MIC]. Our goal is to evaluate identification of MIC by both panoramic radiograph [PAN] and cone-beam computed tomography [CBCT]. *Methods.* 150 cases with bilateral MIC were analyzed. Images of a radiolucent canal, within the trabecular bone, surrounded by a radiopaque cortical bone representing the canal walls, and extending to the anterior portion beyond the mental foramen, were considered by two independent radiologists as being images of MIC. PAN and CBCT of these cases were evaluated by 2 other radiologists at different times. Agreement between results of examination methods was assessed by the Kappa coefficient. The interexaminer and intramethod rates for detection of MIC were analyzed by the McNemar test. Gender, mandible side, examiner, and type of method were analyzed by the generalized estimating equations [GEE] model. *Results.* significant difference between examiners [PAN: *P* = 0.146; CBCT: *P* = 0.749] was not observed. Analysis by GEE model showed no significant difference between genders [*P* = 0.411] and examiners [*P* = 0.183]. However, significant difference was observed for identification in both mandible right side [*P* = 0.001], where the identification frequency was higher, and CBCT method [*P* < 0.001]. *Conclusions.* PAN was not shown to be a safe examination to identify MIC. CBCT should always be used in preoperative planning and to reduce the number of complications in implant surgeries.

## 1. Introduction

Knowledge of the anatomy in the region between the mental foramens is still poorly documented [1] although correct identification of the anatomical structures in this region is important for the success of surgical procedures [2]. In the literature, complications can be found due to anatomical variation in the inferior alveolar nerve because this nerve can extend forming a canal of the incisive nerve, with an extension anteriorly to the mental foramen towards the middle line [1]. The section of nerve in front of the mental foramen and just before its ramification to the incisive nerve can be defined as the anterior loop of the inferior alveolar nerve. Their presence should always be considered when planning a surgery in interforaminal region, especially implant surgery, thus avoiding injury to the nerve and neurosensory disorders [3].

Panoramic radiograph [PAN] is an extraoral radiographic technique widely used by many implantodontists and oral and maxillofacial surgeons. However, the reliability of measurements obtained by this method is low due to distortion and magnification inherent in the technique. Furthermore, images can vary widely as they depend on both operator and position of the patient [4]. PAN accuracy to identify the anterior extension of the mental nerve has been described as being limited [5] besides being poorly documented [6]. On the other hand, cone-beam computed tomography [CBCT] has arrived to replace PAN in implantology because it allows analyzing X-ray images in three-dimensions. However, many dental surgeons use only PAN for the surgery of mandibular implant-supported prosthesis [7], mainly because the anterior region has always been considered relatively safe for this procedure.

Therefore, our goal in this study was to investigate the differences observed between images obtained by PAN and CBCT in the visual assessment of the mandibular incisive canal [MIC].

## 2. Material and Methods

We used exams of 300 unidentified patients, who underwent examination by professional request for diagnostic purposes. They included images from the archives of the School of Dentistry of the institution where this research was conducted, which were obtained by PAN and CBCT.

**Figure 1:**
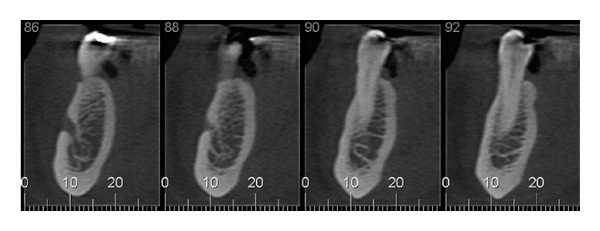
Image defined as being the incisive canal of the mandible [MIC].

Of these 300 exams, we selected 150 cases [75 males and 75 females] whose images in CBCT showed MIC on the right and left sides. Such selection was made by 2 independent radiologists who did not participate in the analysis of images, which was the main object of this study. Images of a radiolucent canal, within the trabecular bone, surrounded by a radiopaque cortical bone representing the canal walls, and extending to the anterior portion beyond the mental foramen were considered as being images of MIC [8] (Figure 1).

Presence of implant in the mandible [which produces artifact in the image], pathological process, and fracture in the mandible were the exclusion criteria.

This study was conducted in accordance with the Declaration of Helsinki; submitted and approved by the research ethics committee of the institution where the research was conducted.

### 2.1. Acquisition of Images

The images of all patients were generated by the same devices. In order to obtain PAN images, the Orthopantomography OP100 device [Instrumentarium; model UC 100-3-1-2, Tuusula, Finland], CR 30-X sensor [Agfa Healthcare, NV, Belgium], and the NX Viewer 2.0.6823 SU2 visualization program [Agfa Healthcare, NV, Belgium, 2007] were used. The respective tomographic images were obtained by a cone-beam tomograph [i-Cat Vision; Imaging Sciences Int. Hatfield, PA, EUA]. The protocol was composed of the following: scanning area: 6 × 16 × 16 cm;tube peak voltage: 120 kV; tube current: 36 mA; exposure time: 40 s; and primary axial reconstruction: 0.25 mm. The visualization program Xoran [v. 3.1.62; Technologies, Ann Arbor, MI, USA] was used.

### 2.2. Analysis of Images

**Figure 2:**
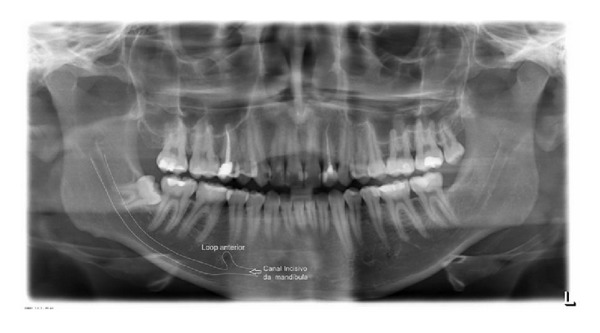
Route of the incisive canal of the mandible [MIC].

The PAN and related CBCT images were analyzed by 2 oral radiologists examiners [minimum experience: 5 years] on two different times: T1 in which they examined the panoramic radiographs confirming or not the presence of MIC image on the right and left sides and T2 in which 1 month later, they proceeded in the same manner in the examination of tomographic images. The examiners were instructed to consider as MIC the images of a radiolucent canal, within the trabecular bone, surrounded by a radiopaque cortical bone representing the canal walls, and extending to the anterior portion beyond the mental foramen (Figure 2), and they could not edit the images. The examiners were not informed that all images of the sample contained MIC or that the sample was divided by gender.

**Figure 3:**
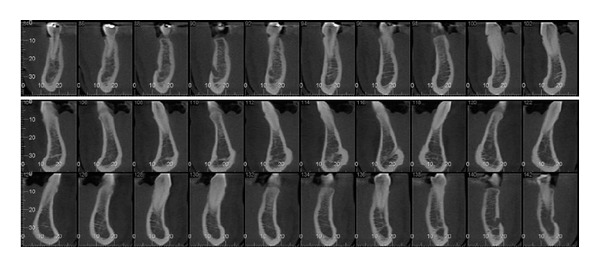
Model of parasagittal sections of the mandible as analyzed by the examiners.

In the CBCT scans (Figure 3), the examiners assessed only parasagittal sections in the region beyond the mental foramen.

All radiographic and tomographic images were examined in a dark room and in the same notebook [Intel 8940 core processor, 2.0 GHz, 2 MB, L3 Cache; Intel HD Graphics; 15.6′′ HD LED LCD monitor, 3 GB memory, Windows 7 operating system], and the examiners might use zoom to magnify the images of interest.

The examiners had to answer yes or no regarding the presence of MIC in the right and left sides of the mandible in the images obtained with both methods.

### 2.3. Statistical Analysis

Agreement between PAN and CBCT as well as between examiners was analyzed by the kappa [*κ*] coefficients of agreement. The percent rates to detect MIC of both interexaminers and intramethods [and between methods] were compared using the McNemar test because these are comparisons within the same group [dependent samples].

A model of generalized estimating equations [GEE; with binomial distribution] was used to assess the effect of gender, side, examiner, and exam type on the probability of detecting MIC. In this model, gender, side, examiner, and method were regarded as independent variables, and detection of MIC was regarded as a dependent variable. This methodology was chosen in order to take into account dependence between assessments of the same patient. In this study, each patient had 6 different data [2 methods, 2 sides, and 2 examiners]. Thus, it was possible to consider all information without the need to summarize these data. All terms of interaction between variables were investigated.

The Statistical Package for Social Sciences (SPSS) [v. 19.0] and R [v. 13.0] programs were used for the calculations.

## 3. Results

In the panoramic radiographs, the MIC was identified in 97 [32.3%; examiner 1] and 82 [27.3%; examiner 2] images of 300 examinations [150 on the left and right sides]. In CBCT, MIC was identified in 271 [90.3%; examiner 1] and 269 [89.7%; examiner 2] of the images. The McNemar test indicated that there was no statistically significant difference between the examiners when they examined the images obtained by the two methods [PAN: *P* = 0.146; CBCT: *P* = 0.749]; that is, there is no evidence that an examiner identified the MIC more frequently than the other did.

Table 1 shows that the degree of agreement [kappa, *κ*] between examiners was low for both PAN [*κ* = 0.262] and CBCT [*κ* = 0.244].

Table 2 presents data for comparison between PAN and CBCT. The values obtained for the coefficients of agreement were very low [some of them were negative], indicating no agreement between both examinations regardless of examiner or side. While examining the percent rates, we found that the low agreement is due to the images identified among those obtained by CBCT but not by PAN. When comparing the methods, the difference found between examiners was statistically significant [*P* < 0.001]; that is, the frequency of identifications of MIC in CBCT images is higher.

Table 3 shows the values obtained utilizing the GEE model to evaluate the influence of gender, side, examiner, and type of examination on the probability of identifying MIC. In this model, the effect of either gender [*P* = 0.411] or examiner [*P* = 0.183] was not observed. However, the probability of identifying MIC was higher [OR = 1.56; CI 95% = 1.21–2.01; *P* = 0.001] on the right side and in the CBCT images [OR = 22.81; CI 95% = 15.16–34.31; *P* < 0.001] when compared to those of PAN.

## 4. Discussion

Among the 300 analyses of images [150 on each side] performed in this study, the examiners did not identify MIC in about 70% of PAN images and 10% of CBCT images. These data confirm the difficulty of the examiners to identify this anatomical structure in PAN images and, therefore, the importance of CBCT for surgical planning in this region. Our results confirm those of Jacobs et al. [9], who identified MIC in 93% of tomographic images examined by them.

Pires et al. [8] also examined 89 CBCT and PAN images and identified MIC in 83.1% of CBCT images and only 11.2% of PAN images. Making measurements on CBCT images of MIC and establishing relationship with side and gender were their priority. However, they found no statistically significant difference between the images regarding these aspects. Otherwise, we herein effectively compared the image methods [radiography versus tomography] by different examiners in order to check the degree of discrepancy between methods and examiners as identification of MIC in PAN images is poorly documented [1, 6].

In our study, not all MIC were identified by the examiners even on CBCT images. Likewise, Parnia et al. [10] have shown that MIC can be identified in 83.3% of tomographic images. In contrast, the mental foramen could be identified in 100% of CBCT images of the same study. It is possible that the reason for the lower rate of the MIC identification by the examiners is that it becomes thinner as follows to middle line [9–11]. In addition, the incisive canal has less cortical bone than the mandibular one [12], which would make its identification difficult in some tomographic sections. Furthermore, in many cases the canal gradually narrows until the neurovascular bundle enters a labyrinth of medullary spaces without strictly forming a canal [13]. Perhaps this is also the reason for the low importance given to the MIC by implantodontists. Specifically in these cases, placement of implant does not seem to cause paresthesia or other undesired effects [14] or even an effect that is recognized by the patients [13].

With regard to identifying MIC by both PAN and CBCT, there was no evidence that one of the examiners identified MIC with a higher or lower frequency than the other. However, our study showed interesting differences between examiners; for example, the coefficient of agreement between them was low in both methods (Table 1). These data explain that MIC is quite identified in CBCT images, although such identification also depends on the experience of each examiner. On the other hand, these differences are especially worrisome regarding identification of MIC in PAN images. In only 43 [14.3%] of 300 images examined, both examiners agreed that MIC was present in the panoramic radiographs (Table 1). Table 2 also shows that MIC was identified in both PAN and CBCT in only 30.2% [examiner 1] and 25% [examiner 2] of the images. In comparing methods, the difference between identification rates was statistically significant for both examiners; that is, the rate for the identification of MIC in images of CBCT is higher than in those of PAN.

Parnia et al. [10] reported values of 1.49 and 1.44 mm for the mean diameter of MIC on the right and left sides, respectively. A number of other measurements, such as distance from MIC to the either lingual or inferior border of the mandible, showed that a slight advantage on the right over the left side is always observed regarding size. In the study by Pires et al. [8], the value for mean length of MIC on the right side is higher than that on the left side, but the difference is not statistically significant. It is not possible to determine whether such differences were determinant for a higher frequency of identification of MIC on the right side compared with the left side as found in our study. However, it is possible that this slight advantage in size has facilitated its identification.

Our study shows both the importance of identifying MIC in planning a surgery in the region between the mental foramens and the difficulty to identify this structure in PAN images, although it almost always is present in those of patients' examinations [14]. Table 3 shows that regardless of gender, side, or examiner, the frequency for identification of MIC with CBCT is higher than that with PAN. However, many dental surgeons still plan surgeries in their patients using only PAN as imaging examination [13, 15], probably because of the high costs of CBCT and the problems of health insurances covering their costs.

Romanos and Greenstein [14] state that the mental foramen and its anterior loop as seen in images of PAN should not be taken exclusively as a basis for planning a surgery because there is a risk of the dental surgeon to hit the incisive nerve and cause a traumatic neuroma. More commonly, installation of implant within the MIC may result in edema of the incisive nerve, which can extend to the mental nerve causing then neurosensory sequel in the lip and mentum. Other authors noticed transient disturbances and discomfort after either installing implant in the interforaminal area or removing bone from the mental region [16]. Another problem is that osteointegration is harmed by migration of soft tissue cells, which adhere around the threads of the implant [10]. Unfortunately, the literature is rich in information on implant loss related to osteointegration. However, studies on implant loss related to either nerve injury or neuropathic pain are rare.

Although numerous studies report inferior alveolar nerve damage during implant placement, few reports in the literature describe sensory disturbances, such as neuropathic pain, related to mandibular incisive nerve damage, which causes difficulty to quantify such episodes. Kütük et al. [17] recently made a retrospective clinical study evaluating the risk of neuropathic pain caused by implant placement in the interforaminal region of the mandible. Fifty-five patients with suspected relationship between MIC and dental implant were included in this study. Computed tomography scans were obtained from 10 patients who had postoperative neuropathic pain. Relationship between dental implant and mandibular incisive nerve was evaluated using a three-dimensional software program and a mandibular incisive nerve perforation by at least 1 implant was observed in all 10 patients. According to the authors, neuropathic pain may occur after implant placement in the interforaminal region due to the perforation of the MIC and nerve. According to the results of this retrospective study, the MIC and nerve perforation should be considered as a complication of implant surgery in the mandibular anterior area.

Moreover, rare cases of acute bleeding are described. A case of brisk, pulsatile bleeding from the anterior MIC, and its management using an active hemostatic matrix was presented by Lee at al. [18]. Probably, the way that the canal gradually narrows until the neurovascular bundle enters a labyrinth of medullary spaces without strictly forming a canal [13] may explain the few hemorrhagic events described.

There is no doubt that PAN underestimates the real presence of MIC. Therefore, identifying the MIC besides identifying the length of the anterior loop of the mental foramen in CBCT images is essential so there are always both safety when placing an implant in the anterior region of the mandible and decrease in the number of postoperative complications [14]. Our study showed that oral radiologists obtain high rates of identification of MIC when CBCT images are used. CBCT should be considered as essential preoperative planning before anterior mandibular implants. Therefore, only use of PAN images for implant placement is not safe.

## Figures and Tables

**Table 1:** Agreement between examiners by type of examination for identification of the mandible incisive canal [MIC].

Type of exams and sides	Identification of the MIC by examiners E1 and E2				Agreement rates [%]	*κ* values∗
	Not identified by both examiners	Identified by both examiners	Identified by E1 but not by E2	Identified by E2 but not by E1		
PAN						
R	77 [51.3]	25 [16.7]	31 [20.7]	17 [11.3]	68.0	0.280
L	87 [58.0]	18 [12.0]	23 [15.3]	22 [14.7]	70.0	0.239

Total [R + L]	164 [54.7]	43 [14.3]	54 [18.0]	39 [13.0]	69.0	0.262

CBCT						
R	1 [0.7]	133 [88.7]	8 [5.3]	8 [5.3]	89.3	0.054
L	8 [5.4]	117 [79.1]	13 [8.8]	10 [6.8]	83.3	0.321

Total # [R + L]	9 [3.0]	250 [83.9]	21 [7.0]	18 [6.0]	86.9	0.244

*Coefficient of agreement; number of identifications [percent rate of identifications in parentheses]. PAN: panoramic radiograph; CBCT: cone-beam computed tomography; R: right side; L: left side; E: examiner. # Two cases of CBCT were not analyzed by E1 and were excluded from the comparison.

**Table 2:** Mandible incisive canal [MIC] as identified by examiners in images of panoramic radiograph [PAN] and cone-beam computed tomography [CBCT].

Examiners and sides	Presence of the MIC according to the examiners				Agreement rates [%]	*κ* values∗
	Absent in both types of exams	Present in both types of exams	Present in PAN but not in CBCT	Present in CBCT but not in PAN		
Examiner 1						
R	7 [4.7]	54 [36.0]	2 [1.3]	87 [58.0]	40.7	0.030
L	15 [10.1]	36 [24.3]	3 [2.0]	94 [63.5]	34.4	0.035

Total # [R + L]	22 [7.4]	90 [30.2]	5 [1.7]	181 [60.7]	37.6	0.037

Examiner 2						
R	6 [4.0]	39 [26.0]	3 [2.0]	102 [68.0]	30.0	−0.009
L	18 [12.0]	36 [24.0]	4 [2.7]	92 [61.3]	36.0	0.037

Total [R + L]	24 [8.0]	75 [25.0]	7 [2.3]	194 [64.7]	33.0	0.014

*Coefficient of agreement; Number of identifications [percent rate of identifications in parentheses]. Abbreviations: PAN: panoramic radiograph; CBCT: cone-beam computed tomography; R: Right side; L: Left side; E: examiner. # Two cases of CBCT were not analyzed by Examiner 1 and were excluded from the comparison.

**Table 3:** Influence of some variables on the probability of identifying the mandible incisive canal [MIC] as calculated by the GEE model.

Independent variables	Odds ratio	CI95%	*P* values
Sides			
R	1.561	1.212–2.009	0.001
L	1∗		

Examiners			
E1	1.238	0.904–1.695	0.183
E2	1∗		

Genders			
M	1.186	0.790–1.781	0.411
F	1∗		

Examinations			
CBCT	22.807	15.162–34.305	<0.001
PAN	1∗		

CI95%: 95% confidence interval; *reference category.

## References

[1] Mraiwa N, Jacobs R, Moerman P, Lambrichts I, van Steenberghe D, Quirynen M (2003). Presence and course of the incisive canal in the human mandibular interforaminal region: two-dimensional imaging versus anatomical observations. *Surgical and Radiologic Anatomy*.

[2] Pommer B, Tepper G, Gahleitner A, Zechner W, Watzek G (2008). New safety margins for chin bone harvesting based on the course of the mandibular incisive canal in CT. *Clinical Oral Implants Research*.

[3] Al-Ani O, Nambiar P, Ha KO, Ngeow WC (2013). Safe zone for bone harvesting from the interforaminal region of the mandible. *Clinical Oral Implants Research*.

[4] Monsour PA, Dudhia R (2008). Implant radiography and radiology. *Australian Dental Journal*.

[5] Jacobs R, Mraiwa N, van Steenberghe D, Sanderink G, Quirynen M (2004). Appearance of the mandibular incisive canal on panoramic radiographs. *Surgical and Radiologic Anatomy*.

[6] Mardinger O, Chaushu G, Arensburg B, Taicher S, Kaffe I (2000). Anatomic and radiologic course of the mandibular incisive canal. *Surgical and Radiologic Anatomy*.

[7] Hu KS, Choi DY, Lee WJ, Kim HJ, Jung UW, Kim S (2012). Reliability of two different presurgical preparation methods for implant dentistry based on panoramic radiography and cone-beam computed tomography in cadavers. *Journal of Periodontal and Implant Science*.

[8] Pires CA, Bissada NF, Becker JJ, Kanawati A, Landers MA (2012). Mandibular incisive canal: cone beam computed tomography. *Clinical Implant Dentistry and Related Research*.

[9] Jacobs R, Mraiwa N, van Steenberghe D, Gijbels F, Quirynen M (2002). Appearance, location, course, and morphology of the mandibular incisive canal: an assessment on spiral CT scan. *Dentomaxillofacial Radiology*.

[10] Parnia F, Moslehifard E, Hafezeqoran A, Mahboub F, Mojaver-Kahnamoui H (2012). Characteristics of anatomical landmarks in the mandibular interforaminal region: a cone-beam computed tomography study. *Medicina Oral, Patologia Oral y Cirugia Bucal*.

[11] Uchida Y, Noguchi N, Goto M (2009). Measurement of anterior loop length for the mandibular canal and diameter of the mandibular incisive canal to avoid nerve damage when installing endosseous implants in the interforaminal region: a second attempt introducing cone beam computed tomography. *Journal of Oral and Maxillofacial Surgery*.

[12] Rosa MB, Sotto-Maior BS, Machado VC, Francischone CE (2013). Retrospective study of the anterior loop of the inferior alveolar nerve and the incisive canal using cone beam computed tomography. *The International Journal of Oral & Maxillofacial Implants*.

[13] Apostolakis D, Brown JE (2013). The dimensions of the incisive canal and its spatial relationship to various anatomical landmarks of the mandible: a study using cone beam computed tomography. *The International Journal of Oral & Maxillofacial Implants*.

[14] Romanos GE, Greenstein G (2009). The incisive canal: considerations during implant placement: case report and literature review. *The International Journal of Oral & Maxillofacial Implants*.

[15] Madrigal C, Ortega R, Meniz C, López-Quiles J (2008). Study of available bone for interforaminal implant treatment using cone-beam computed tomography. *Medicina Oral, Patologia Oral y Cirugia Bucal*.

[16] Wismeijer D, van Waas MAJ, Vermeeren JIJF, Kalk W (1997). Patients' perception of sensory disturbances of the mental nerve before and after implant surgery: a prospective study of 110 patients. *The British Journal of Oral and Maxillofacial Surgery*.

[17] Kütük N, Demirbaş AE, Gönen ZB (2013). Anterior mandibular zone safe for implants. *Journal of Craniofacial Surgery*.

[18] Lee CYS, Yanagihara LC, Suzuki JB (2012). Brisk, pulsatile bleeding from the anterior mandibular incisive canal during implant surgery: a case report and use of an active hemostatic matrix to terminate acute bleeding. *Implant Dentistry*.

